# First detection and characterization of genetically divergent canine calicivirus strains in domestic dogs in China

**DOI:** 10.3389/fvets.2024.1501632

**Published:** 2024-12-13

**Authors:** Sihan Li, Liangyu Chu, Yancheng Zhang, Yaxuan Yu, Guoqing Wang

**Affiliations:** ^1^College of Medicine and Biological Information Engineering, Northeastern University, Shengyang, China; ^2^College of Life and Health Sciences, Northeastern University, Shengyang, China

**Keywords:** canine calicivirus, vesiviruses, companion animals, genetic characterization, phylogenetic analysis

## Abstract

Canine calicivirus (CaCV) belongs to the *Caliciviridae* family, which invades multiple host species. Notably, there are distinct serological and genetic differences between CaCV and other caliciviruses. However, the genome data for only 13 strains of CaCV have been recorded. Moreover, there have been no reports on the CaCV genome in China. To understand the genetic characteristics of CaCV in China, this study tested CaCV in 52 canine nasal swab samples by RT–PCR and finally determined that two samples were positive for this virus. The complete genome sequences of both CaCV strains were obtained through sequencing, with a genomic length of 8,453 bp. The genomic sequences of the two Chinese CaCV strains presented 83.6% nucleotide similarity with each other but 71.6%−90.2% nucleotide similarity with previously reported CaCV strains, indicating that these two CaCV strains were genetically divergent. The subsequent sequence analysis indicated that these two strains possessed unique nucleotide sites in their genomic sequences. Phylogenetic analysis revealed that the Chinese CaCV strains belonged to different evolutionary branches. Overall, the present study provides new knowledge on the prevalence and genetic diversity of CaCV in China.

## 1 Introduction

Vesiviruses have garnered significant attention because of their profound impact on the health of both humans and animals (1). As members of the *Caliciviridae* family, these viruses possess a single-stranded RNA genome and a capsid characterized by distinctive cup-shaped depressions (2). Despite their relatively small size, vesiviruses are resilient and can persist in the environment for extended durations, posing a formidable challenge for disease control (3).

The feline calicivirus (FCV), a prominent member of the *Vesivirus* genus, primarily impacts domestic cats, leading to upper respiratory tract infections (4). Conversely, research on canine calicivirus (CaCV), which is prevalent in dogs kept as pets, is relatively rare. In 1998, Japanese researchers identified a strain of CaCV, derived from a two-month-old dog with recurrent watery diarrhea (5). This CaCV strain exhibited no antigenic or genetic correlation with FCV and was initially categorized as a genuine CaCV within the *Vesivirus* genus (5). Subsequent serological surveys revealed evidence of the virus within canine populations in Japan and Korea, and the complete genomes of analogous viruses have been isolated from dogs in Italy and Switzerland (6–10).

At present, there are only 13 viral sequences available for reference concerning the complete genome sequence of CaCV in the NCBI database (https://www.ncbi.nlm.nih.gov/). Furthermore, reports and strain information concerning CaCV in China are lacking. To clarify the genetic characterization and evolution of CaCV in China, we collected 52 nasal swab samples from dogs in Liaoning in 2024 and examined them for CaCV via RT–PCR. Among these samples, we detected 2 positive cases, and complete genome information for two strains of CaCV was obtained and subsequently analyzed.

## 2 Materials and methods

### 2.1 Sample collection and preparation

To further characterize CaCV within canine populations, a comprehensive sampling strategy was implemented. A total of 52 nasal swab samples were collected from dogs in Liaoning Province in 2023 (Supplementary Table 1). Prior to sample collection, the owners were informed about the study's objectives and provided consent for their pets to participate. After collection, these samples were immediately stored at −80°C for further use.

### 2.2 RNA extraction, virus detection and screening

Total RNA was extracted using TRNzol Universal (TianGen, Beijing, China) in accordance with the manufacturer's instructions. The extracted RNA was subsequently subjected to reverse transcription polymerase chain reaction with a FastKing cDNA First Strand Synthesis Kit (TianGen, Beijing, China) with a random primer serving as the reverse transcription primer. To detect CaCV, PCR amplification was performed using the specific primers p289/p290 and 493F/526R (11). Additionally, the universal primers 3171F/3361R were developed on the basis of the conserved sequences of the CaCV genome, and the PCR conditions were fine-tuned to guarantee both specificity and sensitivity (Supplementary Table 2).

### 2.3 Whole genome sequencing

The viral genome was acquired through gap-filling PCR, which employs universal primers designed to target various sections of the CaCV genome. The primers utilized for genome sequencing are detailed in Supplementary Table 2. DNA fragments of the anticipated size, as visualized on a 1% agarose gel following electrophoresis, were subsequently purified. These purified fragments were then inserted into the pCloneEZ-blunt vector (Clone Smarter, USA), sequenced (BGI, China), and introduced into *E. coli DH5*α competent cells (TianGen, Beijing, China) via transformation for further sequencing.

### 2.4 Sequencing and phylogenetic analyses

To gain insights into the genetic diversity and evolutionary relationships of CaCV among different canine populations, the raw sequencing data were assembled and processed using SeqMan 7.1.0. The data were subsequently aligned with those of other CaCV strains using BioEdit 5.0.7.0. Following estimation via “Find Best DNA Models,” a phylogenetic tree was established using MEGA 6.0, employing the maximum likelihood method with the LG+G+I model, which is based on the bootstrap values of 1,000 replicates.

### 2.5 Recombinant analysis

In addition, a standard similarity plot analysis was conducted using the non-structural polyprotein, VP1, hypothetical protein of Chinese strains as queries for comparison with the other CaCV strains. This was achieved using the SimPlot v.3.5.1 software, with a window size of 200 bp and a step size of 20 bp.

To detect potential recombination events, CaCV sequences were subjected to seven distinct methods (RDP, GENECONV, Chimera, MaxChi, BootScan, SiScan, and 3Seq) via the Recombination Detection Program (RDP) version 5.05. an event with a *p* value of 0.01 and a recombination score of >0.6 was considered a possible recombination event.

### 2.6 GenBank accession numbers

The GenBank accession numbers for CaCV strains R24032707 and R24032708 are PQ219770 and PQ219771, respectively.

## 3 Results

In this research, we successfully identified two CaCV-positive samples (3.8%, 2/52) among 52 canine nasal swab samples using three pairs of detection primers. Through gap-filling PCR, sequencing, sequence alignment, and sequence assembly, the full genomic sequences of the two CaCV strains were successfully obtained and named R24032707 and R24032708. The two CaCV strains had a genomic length of 8,453 bp, with ORF1 CDS lengths of 5,796 bp and 5,802 bp, ORF2 lengths of 2,073 bp and 2,079 bp, and ORF3 lengths of 405 bp each. Compared with the sequence of the reference strain Attila, the R24032708 strain contained an extra insertion of an extra glycine (G) and arginine (R) at positions 830 and 1,580 within ORF1. Conversely, the R24032707 strain featured the addition of a serine (S) and an aspartic acid (N) at the 442 and 443 positions of ORF2, respectively.

The aim of this study was to clarify the genetic characterization of CaCV by comparing the nucleotide similarity of R24032707, R24032708, and other CaCVs published in the NCBI database (Tables 1, 2). The genome sequence homologies for strain R24032707 across ORF1, ORF2, and ORF3 were 71.6%−90.2%, 73.7%−92.4%, 67.1%−85.7%, and 23.5%−91.6%, respectively. For strain R24032708, the corresponding homologies for ORF1, ORF2, and ORF3 were 72.7%−82.9%, 74.4%−83.1%, 66.8%−85.2%, and 24.2%−86.9%, respectively. Additionally, the Chinese strains presented genome sequence homologies of 83.6% for ORF1, 83.4% for ORF2, 82.5% for ORF3, and 91.6% for ORF1, ORF2, and ORF3, respectively. Among them, the whole-genome nucleotide homology of R24032707 was the most similar to that of the Belgian strain Geel, reaching 90.2%, whereas R240327078 showed the highest similarity to the USA strain Attila, at 83.7%.

**Table 1:** The nucleotide similarities between R24032707 and other CaCV strains.

**R24032707**	**Complete genome**	**ORF1**	**ORF2**	**ORF3**
R24032708	83.6%	83.4%	82.5%	91.6%
CaCV/NC_004542.1/Canine-vesivirus/2018/JAP	72.5%	74.5%	66.7%	70.1%
CaCV/JN204722.1/Bari/2007/ITA	89.7%	91.6%	81.4%	84.4%
CaCV/MF978270.1/CU/296/2015/USA	89.9%	92.2%	80.1%	85.7%
CaCV/MK290748.1/Attila/2014/FRA	90.0%	92.4%	79.7%	85.2%
Calicivirus/GQ475301.1/Allston/2009/USA	89.4%	91.7%	81.4%	86.7%
Calicivirus/GQ475302.1/Allston/2008/USA	89.5%	91.7%	81.6%	86.7%
Calicivirus/GQ475303.1/Geel/2008/BEL	90.2%	91.8%	83.8%	24.2%
CaCV/MF327135.1/A128T/1968/USA	72.3%	73.9%	67.0%	24.7%
CaCV/MF327134.1/3-68/1968/USA	87.9%	91.1%	79.2%	23.5%
CaCV/MF327137.1/W191R/1973/USA	88.2%	90.7%	85.7%	24.4%
CaCV/MF327136.1/L198T/1968/USA	71.6%	73.7%	67.1%	26.4%
Calicivirus/AY343325.2/2117/2003/CHO	82.9%	85.5%	79.5%	24.4%
FCV/OR000445.1/W109-1443/2024/CHN	48.5%	52.1%	47.5%	34.6%

**Table 2:** The nucleotide similarities between R24032708 and other CaCV strains.

**R24032708**	**Complete genome**	**ORF1**	**ORF2**	**ORF3**
R24032707	83.6%	83.4%	82.5%	91.6%
CaCV/NC_004542.1/Canine-vesivirus/2018/JAP	73.6%	75.3%	67.2%	70.9%
CaCV/JN204722.1/Bari/2007/ITA	82.7%	82.5%	82.2%	85.4%
CaCV/MF978270.1/CU/296/2015/USA	82.9%	83.0%	80.2%	86.9%
CaCV/MK290748.1/Attila/2014/FRA	83.1%	83.1%	81.1%	86.4%
Calicivirus/GQ475301.1/Allston/2009/USA	82.6%	82.9%	80.5%	87.2%
Calicivirus/GQ475302.1/Allston/2008/USA	82.6%	82.8%	80.7%	87.2%
Calicivirus/GQ475303.1/Geel/2008/BEL	82.5%	82.8%	81.3%	24.2%
CaCV/MF327135.1/A128T/1968/USA	73.1%	74.4%	66.8%	24.9%
CaCV/MF327134.1/3-68/1968/USA	81.8%	81.9%	85.2%	24.7%
CaCV/MF327137.1/W191R/1973/USA	80.3%	81.3%	81.3%	26.2%
CaCV/MF327136.1/L198T/1968/USA	72.7%	74.6%	66.8%	26.4%
Calicivirus/AY343325.2/2117/2003/CHO	77.0%	77.5%	79.9%	25.4%
FCV/OR000445.1/W109-1443/2024/CHN	47.8%	51.0%	47.5%	34.6%

In addition, no potential recombination events within CaCV strains were identified after systematic analyses were performed.

In the analysis of amino acid homology among CaCV isolates, this study revealed that the amino acid homology of R24032707 with the non-structural polyprotein and VP1 proteins of other CaCV isolates was 79.5%-98.6% and 70.8%-94.9%, respectively; the amino acid homology of R24032708 with the non-structural polyprotein and VP1 proteins of other CaCV isolates was 79.5%-90.7% and 72.4%-93.1%, respectively (Supplementary Tables 3, 4). Moreover, a comparative analysis with other CaCV amino acids revealed that the FRAES region (capsid cleavage site), the PPG amino acid site, and the 7-amino-acid CaCV-specific insertion (N/S/K)(S/A/T)IKS(D/S/Q)(I/V) of R24032707 and R24032708 are also conserved (8, 12).

**Figure 1:**
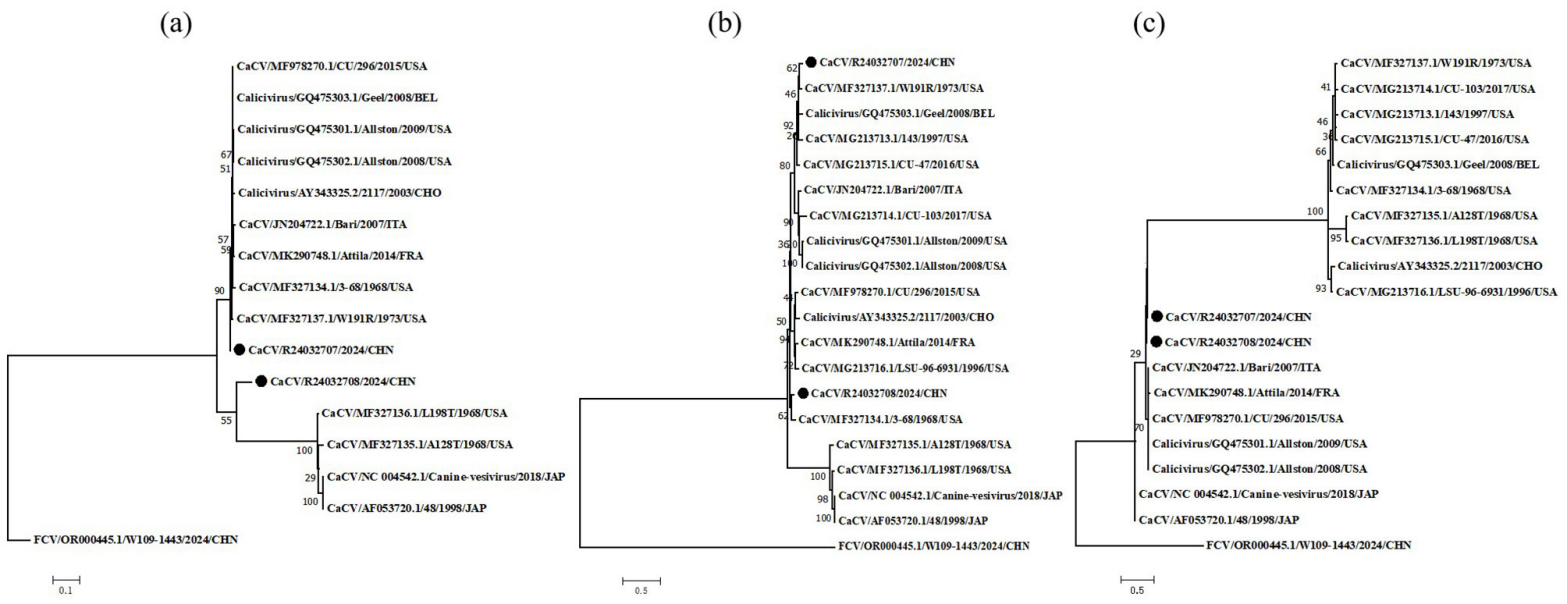
Phylogenetic analysis of CaCV based on the nonstructural polyprotein, VP1 and hypothetical protein. Phylogenetic analysis of canine caliciviruses based on complete amino acid sequences of nonstructural polyprotein **(A)**, b major capsid protein VP1 **(B)** and hypothetical protein **(C)**. CaCV, canine calicivirus; FCV, feline calicivirus. The solid black circle represents the viral strain sequences obtained from this study.

To clarify the origin of these viruses, we conducted phylogenetic analyses of the non-structural polyprotein, VP1 and hypothetical protein (Figure 1). The phylogenetic tree results revealed that the nonstructural polyprotein, VP1, and the hypothetical protein of CaCV could be divided into two major evolutionary branches. The L98T, A28T, 48 and canine-vesivirus strains were clustered together in non-structural polyprotein and VP1. In the phylogenetic tree of non-structural polyproteins, the R24032708 strain was genetically distinct from other CaCV strains and was classified into a separate branch, whereas the R24032707 strain was positioned within another major evolutionary branch. Conversely, within the VP1 phylogenetic tree, Chinese strains were clustered into the same major branch, with the R24032708 strain being more closely related to the USA strain 3–68 and the R24032707 strain being grouped into a minor branch alongside the USA strain W191R. In the phylogenetic tree of the hypothetical protein, Chinese strains are divided into the same small evolutionary branch. An analysis of the non-structural polyprotein and hypothetical protein evolutionary branches revealed that the R24032707 and R24032708 strains were slightly more evolutionarily distant from the strains.

## 4 Discussion

Although some reports have revealed a potential link between CaCV and canine diarrhea, other studies have shown that the virus can be detected in canine nasal swab samples (8, 10). This study also revealed CaCV in canine nasal swab samples. The absence of anal swab testing may affect the detection rate of this virus. Nonetheless, during the sample collection process, the overwhelming majority of pet owners were hesitant to permit us to collect anal swabs. Therefore, the exact pathogenicity of CaCV still requires further exploration in future research. The high similarity between the complete genomes and the ORF1, ORF2, and ORF3 sequences of R24032707 and R24032708 suggests a recent common ancestor or ongoing transmission between the two strains. However, the differences observed in their amino acid sequences and phylogenetic positions indicate that they have diverged to some extent, likely due to independent evolutionary pressures. The insertion of amino acids into ORF1 and ORF2 of R24032707 and R24032708, respectively, may have a significant effect on the antigenicity and pathogenicity of these strains (13).

The conservation of amino acids within the FRAES region, the PPG motif, and the 7-amino-acid insertion provides robust evidence supporting the critical role these domains play in maintaining the structural integrity and functional capacity of the capsid protein [17]. The retention of these sequences across diverse CaCV strains highlights their importance in upholding the viral life cycle and infectivity (14).

The results of the phylogenetic analyses suggest that the two CaCV strains, R24032707 and R24032708, share a common ancestry but have diverged significantly over time, leading to distinct evolutionary paths. The non-structural polyprotein phylogenetic tree indicates that R24032708 has evolved independently, forming a unique evolutionary branch separate from the larger group that includes R24032708. These findings suggest that R24032708 may have undergone specific adaptations or mutations that set it apart from other CaCV strains. Conversely, the VP1 phylogenetic tree revealed that both the R24032707 and R24032708 strains belong to the same major evolutionary branch, but they are differentiated into smaller subbranches. The proximity of R24032708 VP1 to the 368 strain suggests a closer evolutionary relationship in this particular gene, potentially indicating a shared ancestor or similar selective pressures. In contrast, R24032707 VP1 clusters with the W191 strain, suggesting a different evolutionary trajectory. However, genetic information about the CaCV strain is still relatively limited, and the specific viral genetic situation has not been clearly defined.

In conclusion, genetic characterization and phylogenetic analyses of the CaCV strains R24032707 and R24032708 have provided valuable insights into their origins, evolution, and potential impact on viral function. Further studies are needed to elucidate the functional consequences of the observed amino acid insertions and to investigate the epidemiology and transmission dynamics of these viruses in their natural hosts. Understanding the genetic diversity and evolution of CaCV is crucial for developing effective diagnostic tools, vaccines, and antiviral strategies to combat this emerging pathogen.

## Data Availability

The datasets presented in this study can be found in online repositories. The names of the repository/repositories and accession number(s) can be found in the article/Supplementary material.
