# Supplementary material for: First detection and characterization of genetically divergent canine calicivirus strains in domestic dogs in China
Source: Front Vet Sci. 2024 Dec 13;11:1501632. doi: 10.3389/fvets.2024.1501632 (PMC11671504; doi:10.3389/fvets.2024.1501632)
Supplement: Supplementary file 1 [file Data_Sheet_1.zip › Supplementary Files/Supplementary table 2.docx]

**Supplementary table 2. Primers used for virus detection and genome sequencing**

| Primer | Primer sequence (5′ to 3′) | Sense | Target sequence size (bp) | Use |
| --- | --- | --- | --- | --- |
| 493F^a^ | GGTTTGCCATCTGGCATGCCGCTA | + | 689 bp | Detection |
| 526R^b^ | AGCCATVGCTCARTTCTCAAACAC | - |  |  |
| p290 | GATTACTCCAAGTGGGACTCCAC | + | 330 bp | Detection |
| p289 | TGACAATGTAATCATCACCATA | - |  |  |
| 3171F | GTGGATTCAGRTTTGGATCCATT | + | 190 bp | Detection |
| 3361R | GCTTCACTCCTWGGCTTRTA | - |  |  |
| 07/08-1F | GTTAAYKAGAAATGGCTT | + | 1657 bp | Sequencing |
| 07-1657R | ACTTCAGTGAGGCCAATA | - |  |  |
| 07-1572F | CAGAAATTAGCCGCAATGT | + | 1675 bp | Sequencing |
| 07-3246R | GGAACTCACGGAGTATTTCA | - |  |  |
| 07-3138F | TTGATTCGTGCTGGCG | + | 1528 bp | Sequencing |
| 07-4665R | GGTGGTTCATGAGAATCTG | - |  |  |
| 07-4532F | AACACATCTTGGAATGATTG | + | 1596 bp | Sequencing |
| 07-6172R | AAGTAAGAGACAGAGCTA | - |  |  |
| 07-6033F | GGGATTGAAGAGTACATC | + | 1645 bp | Sequencing |
| 07-7677R | TATCGTAGACGTCCTGAGC | - |  |  |
| 07-7421F | GGGAAACTTCACACAGAGT | + | 1033 bp | Sequencing |
| 07-8453R | CCAATTGTTTTGCCAAATA | - |  |  |
| 07/08-1F | GTTAAYKAGAAATGGCTT | + | 1756 bp | Sequencing |
| 08-1756R | GGATCAAGAGTTTTAAGCTT | - |  |  |
| 08-1557F | GGAATAACCGCAATACAG | + | 1874 bp | Sequencing |
| 08-3430R | TCATCGTACTCGTCATCAGA | - |  |  |
| 08-3281F | TTGATTCGTGCTGGCG | + | 1624 bp | Sequencing |
| 08-4904R | GTCCATGTTGATTCCGACTT | - |  |  |
| 08-4666F | ACCTTGCAGACAGATGGCAA | + | 1538 bp | Sequencing |
| 08-6248R | AAGTGACGGATCCATC | - |  |  |
| 08-6127F | CTTTCACCTACCCTAT | + | 1707 bp | Sequencing |
| 08-7833R | CCATTCCCACGTAGCTGGC | - |  |  |
| 08-7672F | ACGACATTGGGCCGGAAGCCT | + | 827 bp | Sequencing |
| 07/08-8453R | CCAATTGTTTTGCCAAATA | - |  |  |

Abbreviation: ^a^ F, forward primer ^b^ R, reverse primer
