# Supplementary material for: First detection and characterization of genetically divergent canine calicivirus strains in domestic dogs in China
Source: Front Vet Sci. 2024 Dec 13;11:1501632. doi: 10.3389/fvets.2024.1501632 (PMC11671504; doi:10.3389/fvets.2024.1501632)
Supplement: Supplementary file 1 [file Data_Sheet_1.zip › Supplementary Files/Supplementary table 3.docx]

**Supplementary table 3. The amino acid similarities of R24032707 and other CaCV strains**

| R24032707 | Nonstructural polyprotein | VP1 |
| --- | --- | --- |
| R24032708 | 90.6% | 89.9% |
| CaCV/NC_004542.1/Canine-vesivirus/2018/JAP | 80.3% | 70.6% |
| CaCV/JN204722.1/Bari/2007/ITA | 98.4% | 89.4% |
| CaCV/MF978270.1/CU/296/2015/USA | 98.7% | 88.1% |
| CaCV/MK290748.1/Attila/2014/FRA | 98.5% | 87.9% |
| Calicivirus/GQ475301.1/Allston/2009/USA | 98.3% | 88.1% |
| Calicivirus/GQ475302.1/Allston/2008/USA | 98.1% | 88.5% |
| Calicivirus/GQ475303.1/Geel/2008/BEL | 98.6% | 92.1% |
| CaCV/MF327135.1/A128T/1968/USA | 79.7% | 71.2% |
| CaCV/MF327134.1/3-68/1968/USA | 97.3% | 87.2% |
| CaCV/MF327137.1/W191R/1973/USA | 96.2% | 94.9% |
| CaCV/MF327136.1/L198T/1968/USA | 79.5% | 70.8% |
| Calicivirus/AY343325.2/2117/2003/CHO | 92.4% | 88.3% |
| FCV/OR000445.1/W109-1443/2024/CHN | 50.6% | 36.2% |
